# Pharmaceutical Characterization and In Vivo Evaluation of Orlistat Formulations Prepared by the Supercritical Melt-Adsorption Method Using Carbon Dioxide: Effects of Mesoporous Silica Type

**DOI:** 10.3390/pharmaceutics12040333

**Published:** 2020-04-08

**Authors:** Heejun Park, Kwang-Ho Cha, Seung Hyeon Hong, Sharif Md Abuzar, Seungyeol Lee, Eun-Sol Ha, Jeong-Soo Kim, In-Hwan Baek, Min-Soo Kim, Sung-Joo Hwang

**Affiliations:** 1 College of Pharmacy, Pusan National University, 63 Busandaehak-ro, Geumjeong-gu, Busan 46241, Korea; pharmacy4336@pusan.ac.kr (H.P.); edel@pusan.ac.kr (E.-S.H.); 2 Yonsei Institute of Pharmaceutical Sciences & College of Pharmacy, Yonsei University, 85 Songdogwahak-ro, Yeonsu-gu, Incheon 21983, Korea; horizon0712@gmail.com (K.-H.C.); shhongmartin@naver.com (S.H.H.); sumonzar@gmail.com (S.M.A.); qwer21244@naver.com (S.L.); 3 Dong-A ST Co. Ltd., Giheung-gu, Yongin, Gyeonggi 446-905, Korea; ttung2nd@naver.com; 4 College of Pharmacy, Kyungsung University, 309, Suyeong-ro, Nam-gu, Busan 48434, Korea; baek@ks.ac.kr

**Keywords:** orlistat, mesoporous silica, supercritical melt-adsorption, crystallinity, in vivo evaluation

## Abstract

Orlistat, an anti-obesity drug, has two critical issues—the first is its low efficacy due to low water solubility and the second is side effects such as oily spotting due to its lipase inhibition. The present study was designed to propose a solution using a formulation with mesoporous silica to simultaneously overcome two issues. Orlistat was loaded onto mesoporous silica by the supercritical melt-adsorption (SCMA) method, using carbon dioxide (CO_2_). Various types of mesoporous silica were used as adsorbents, and the effects of the pore volume, diameter and particle size of mesoporous silica on the pharmaceutical characteristics were evaluated by various solid-state characterization methods and in vitro and in vivo studies in relation to pharmacological efficacy and the improvement of side effects. The results showed that the pore volume and diameter determine loadable drug amount inside pores and crystallinity. The dissolution was significantly influenced by crystallinity, pore diameter and particle size, and the inhibition of lipase activity was in proportion to the dissolution rate. In vivo studies revealed that the serum triglyceride (TG) concentration was significantly decreased in the group administered amorphous orlistat-loaded Neuisilin^®^UFL2 with the highest in vitro dissolution rate and lipase activity inhibition in comparison to the commercial product. Furthermore, oily spotting tests in rats revealed that undigested oil was adsorbed onto mesoporous silica after orlistat was released in the gastro-intestinal tract, and it correlated with in vitro result that oil adsorption capacity was dependent on the surface area of empty mesoporous silica. Therefore, it was concluded that mesoporous silica type plays a major role in determining the pharmaceutical characteristics of orlistat formulation prepared using SCMA with CO_2_ for improving the low solubility and overcoming the side effects.

## 1. Introduction

**Figure 1:**
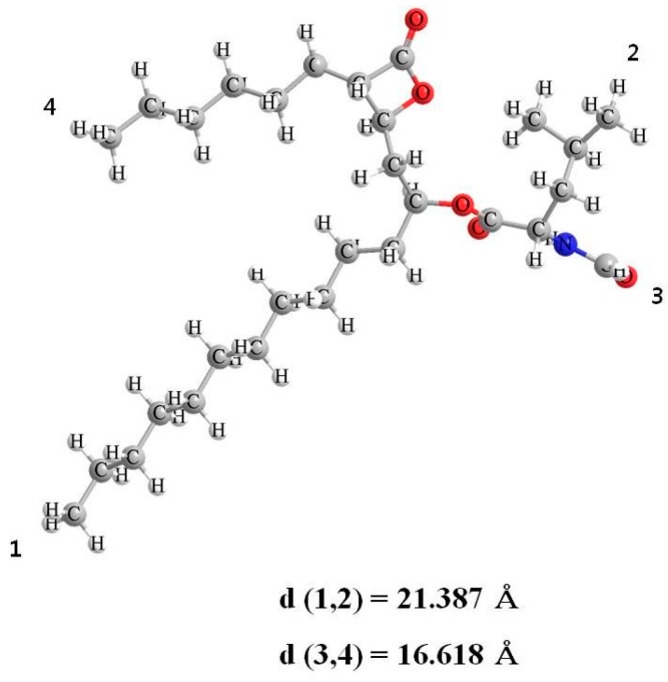
Chemical structure of orlistat.

Orlistat (tetrahydrolipstatin), derived from lipstatin, a natural product of *Streptomyces toxyricini*, is a covalent inhibitor of digestive lipases (Figure 1) [1,2]. Orlistat inhibits the hydrolysis of dietary triglycerides (TG) by covalently blocking the lipase active site, and thus reduces the subsequent intestinal absorption of the lipolysis products, such as monoglycerides and free fatty acids. It acts locally in the gastrointestinal tract when administrated with a meal [3]. The commercial product, Xenical^®^ (Roche Pharmaceuticals, Nutley, NJ, USA), was approved by the Food and Drug Administration (FDA) in 1999 and it was switched to over the counter (OTC) in 2008 [4,5,6]. It has been widely used as an anti-obesity drug all over the world and many clinical studies have shown that orlistat has positive pharmacological efficacy such as cholesterol and TG reduction as well as weight loss [7]. However, the use of orlistat has two major issues that need to be overcome. The first is its low solubility. Orlistat is a biopharmaceutics classification system class II drug with low-solubility and high-permeability [8]. Thus, the slow dissolution rate of orlistat limits its pharmacological activity [9,10,11]. Another significant issue is the side effects associated with the use of orlistat [12]. In most clinical studies, the side effects of orlistat, such as oily spotting, fecal urgency, fatty/oily stool, oily evacuation, fecal incontinence, increased defecation, diarrhea, and abdominal pain were observed [13]. Several studies have been carried out in an attempt to find ways to reduce these side effects, such as: (i) use of a surfactant to stabilize the oil/water interface in order to prevent coalescence of the oil emulsion in the colon; (ii) enhancement of water viscosity in the colon to reduce the intensity and frequency of droplet-droplet interactions, thereby reducing the probability of coalescence; (iii) physical absorption of oil by a lipophilic compound; (iv) increasing the natural stool mass by facilitating bacterial growth in the colon [14,15,16,17,18]. The side effects are caused by the mechanism by which orlistat inhibits lipase.

Recently, the application of mesoporous silica as a drug carrier has attracted much attention. Mesoporous materials are a class of nanoporous materials with pores sizes between 2 and 50 nm [19]. Because of its characteristics such as stable mesoporous structure, uniform and tunable pore size, high total pore volume, high adsorption capacity, non-toxic nature, and biocompatibility, mesoporous silica is a suitable core material for drug delivery [20,21,22,23,24]. Several studies have been carried out to enhance the dissolution of the guest drug using mesoporous silica. Several factors, such as the pore diameter and shape, particle size, and surface area of mesoporous silica and the nature of the host-guest chemical interaction, have been found to affect the drug dissolution profile [25,26,27,28,29,30,31]. To prepare solid dispersion formulations of drug-loaded adsorbents, conventional processes such as solvent evaporation, spray drying, and melt adsorption have been used [32]. However, recently, a melt-adsorption process using supercritical carbon dioxide (SC-CO_2_) has been introduced by some researchers as a more efficient way to load drugs onto adsorbents [33,34,35,36,37]. SC-CO_2_ is the most widely used supercritical fluid because of its mild critical conditions, nontoxicity, non-flammability, and low cost, making it an ideal substitute for organic solvents [38,39,40,41]. Especially, SC-CO_2_ can lower the melting temperature and viscosity of materials via its low viscosity and high diffusivity, thus it has been used for the supercritical melt-adsorption (SCMA) method [40,41,42,43,44,45,46,47,48]. 

The purpose of this study is to use the following principles to simultaneously overcome the two major problems associated with orlistat: (i) enhance the dissolution and pharmacological effect of orlistat by producing an amorphous dispersion formulation with an increased surface area by loading orlistat onto the adsorbent pore using an efficient preparation method, (ii) reduce side effects, such as fatty/oily stool and oily spotting, by adsorption of free oil onto empty mesoporous silica after the release of the adsorbed orlistat. In this study, orlistat was loaded onto mesoporous silica via the SCMA method using CO_2_ to improve its dissolution rate and to reduce side effects. Various types of mesoporous silica adsorbents were used, and the effects of the shape, size, and pore diameter of mesoporous silica on the physicochemical properties and in vivo pharmacological efficacy were evaluated. In addition, for all the adsorbents used, the effects of drug mass ratios were also evaluated. Differential scanning calorimetry (DSC), powder X-ray diffraction (PXRD), scanning electron microscopy (SEM), and analyses of specific surface area analysis, total pore volume and pore diameter analyses were used for solid-state characterization. In vitro dissolution, lipase inhibition, and oil adsorption tests were conducted to select a desired formulation to be used in the animal experiments for each adsorbent. Furthermore, changes in the TG concentration and fat excretion after the oral administration of orlistat formulations were evaluated in rat and mice models, respectively, to determine the in vivo pharmacological effects. To evaluate and compare the improvement in side effects, oily spotting tests were carried out in rats.

## 2. Materials and Methods 

### 2.1. Materials

Orlistat was obtained from Biocon Co., Ltd. (Bangalore, India). Neusilin^®^UFL2 and Neusilin^®^US2 were purchased from Fuji Chemical Industry Co., Ltd. (Toyama, Japan). CO_2_ (99.99% purity) was supplied by Hanmi Gas Co. Ltd. (Daejeon, Korea). MCM-41 (Mobile Crystalline Material), tetraethyl orthosilicate, triblock copolymer [poly(ethylene glycol)_20_–poly(propylene glycol)_70_–poly(ethylene glycol)_20_, 1,2,3-trimethylbenzene (1,2,3-TMB), Solutol HS15 (polyoxyl 15 hydroxystearate), *p*-nitrophenylpalmitate (*p*-NPP), lipase from porcine, and sodium taurodeoxycholate were obtained from Sigma-Aldrich (St. Louis, MO, USA). Sodium deoxycholate was obtained from Acros Co., Ltd. (Morris, NJ, USA). Phosphoric acid, ethanol, olive oil, tris(hydroxymethyl)aminomethane, sodium acetate, l-(+)-tartaric acid, cyclohexane, and propan-2-ol were purchased from Samchun Pure Chemical Co., Ltd. (Pyeongtaek, Korea). Hydrochloric acid and sodium lauryl sulfate (SLS) were purchased from Ducsan Co., Ltd. (Ansan, Korea). Sodium tartrate was obtained from Shimakyu’s Pure Chemical Co. Ltd., (Osaka, Japan). Hydroxypropylmethyl cellulose 2910 (HPMC 2910) was obtained from Shin-Etsu Chemical Co., Ltd. (Tokyo, Japan).

### 2.2. Synthesis of Mesoporous Silica Santa Barbara Amorphous (SBA-15)

The silica SBA-15 was synthesized as described by Jana et al. [49]. The synthesis process was performed by the hydrothermal method at 100 °C, using tetraethyl orthosilicate, hydrochloric acid (35.0–37.0), water, triblock copolymer [poly(ethylene glycol)_20_–poly(propylene glycol)_70_–poly(ethylene glycol)_20_], and an auxiliary chemical, 1,2,3-trimethylbenzene (1,2,3-TMB). Jana et al. reported that the pore diameter of silica SBA-15 increased with the addition of the auxiliary chemical. Based on this report, silica SBA-15 adsorbents, with two different pore sizes, were prepared at two molar gel compositions of SiO_2_:HCl:triblock copolymer:1,2,3-TMB:H_2_O = 1.0:0.43:0.00069:0 and 2:5.8. SBA-15 with large pores was called SBA-15-LP in this study. In the synthesis process, the triblock copolymer was first dissolved in deionized water (DW) while stirring. The 1,2,3-TMB was added to the previously prepared mixture, and then stirred vigorously at room temperature. The temperature of the resultant solution was increased to 50 °C; the solution stirred for another 5 min, and then cooled down to room temperature. Tetraethyl orthosilicate was added to this solution and mixed. For gel formation, HCl was added and the mixture was stirred for 20 h at 35 °C. The formed gel was heated at 100 °C under autogenous pressure in an autoclave for 24 h. The solid product was then separated by filtration under vacuum and washed with large amounts of warm DW. After drying at 100 °C, it was calcined in air at 557 °C for 8 h.

### 2.3. Supercritical Melt-Adsorption Process Using CO_2_

Orlistat-loaded mesoporous silica was prepared by the SCMA method using SC-CO_2_, as described in our earlier report, except that a vessel of 60 mL volume was used [34]. The various types of mesoporous silica used in this study are shown in Table 1. The compositions of the prepared powders, containing different ratios of orlistat and mesoporous silica, are also shown in Table 1. Orlistat and mesoporous silica were weighed accurately, and the initial mixing was done using a tumble mixer. The initial mixture was transferred to the bottom of a 60 mL high-pressure vessel and the vessel was sealed. To reach the desired pressure, SC-CO_2_ was delivered inside the vessel using a high pressure syringe pump (ISCO, Model 260D, Lincoln, NE, USA) through a pre-heated and pressurized container. A heat circulator was used to maintain a constant temperature inside the vessel. The pressure and temperature were fixed at 318.15 K and 10 MPa, respectively, and preliminary studies confirmed that orlistat melted under this condition. To provide sufficient time for the adsorption of the melted orlistat onto pores of mesoporous silica, the supercritical system was maintained at a constant condition for 90 min. After completion of the adsorption process, the vessel was depressurized for approximately 30 min. The prepared powder samples were collected from the vessel.

### 2.4. Solid-State Characterization

#### 2.4.1. Measurements of Specific Surface Area, Total Pore Volume and Pore Diameter

The specific surface area, pore diameter, and total pore volume were measured in a relative pressure (P/P_0_, P_0_ = saturation vapor pressure) range from 10^−4^ to 1 by nitrogen adsorption-desorption studies at −196 °C, using a surface area and pore-size analyzer (ASAP 2010, Micromeritics, Norcross, GA, USA). Before the measurement, the sample powders in tubes were degassed at 25 °C (below melting point of orlistat) for at least 24 h under nitrogen gas purge using a FlowPrep 060 degasser (Micromeritics, GA, USA) until the mass of the sample reaches plateau. The surface area was determined based on the Brunauer–Emmett–Teller (BET) theory using the software provided. The average pore diameter and total pore volume were obtained from the adsorption branches of the isotherms using the Barrett–Joyner–Halenda (BJH) approach. The selected data obtained range from 10^−4^ to 1 was used for the BET and BJH approaches.

#### 2.4.2. Differential Scanning Calorimetry

The DSC analysis was performed using the DSC S-650 instrument (Scinco Co. Ltd., Seoul, Korea). The accurately weighed samples (2–4 mg) were heated in hermetically-sealed aluminum pans, with a heating rate of 10 °C/min, under nitrogen purge and a flow rate of 40 mL/min. An empty sealed pan was used as the reference sample. The relative crystallinity (RC), compared to that of the raw material, was obtained from the following Equation (1) [50]:

RC (%) = (ΔH_sample_/ΔH_raw_) × 100 (1)

where ΔH_raw_ and ΔH_sample_ are the measured melting enthalpies (J/g) of raw orlistat and SCMA processed sample, respectively. The detection of peak temperature and integration of melting peaks for the measurement of melting enthalpy were performed using the Infinity Pro software (Scinco Co. Ltd., Korea).

#### 2.4.3. Powder X-ray Diffraction

PXRD patterns of each pure material and all the SCMA-processed samples containing varying proportions of orlistat in formulation were recorded using an X-ray diffractometer (Rigaku, D/max-ⅢC, Tokyo, Japan), with Ni-filtered Cu-Kα line as the source of radiation. The diffraction pattern was measured with a voltage of 40 kV and a current of 45 mA over a 2θ range of 5°–50°, using a step size of 0.05° at a scanning speed of 5°/min.

#### 2.4.4. Scanning Electron Microscopy

SEM (JSM-7000F, JEOL, Tokyo, Japan) was employed for the morphological analysis. The samples were evenly placed on a metal sample mount using conductive carbon adhesive tape. After coating with a thin layer of gold, the morphology and appearance of the samples were examined at an accelerating voltage of 1 or 5 kV.

### 2.5. In-Vitro Dissolution Test

The dissolution tests were conducted in 900 mL of DW, containing 1% SLS, using the USP II apparatus with VK 7000 dissolution testing station and VK 750d heater/circulator (Vankel, Cary, NC, USA). Powder samples (equivalent of 60 mg of orlistat) were placed in the dissolution medium, which was maintained at 37 ± 0.5 °C and stirred at 75 rpm. Each sample (2 mL) was withdrawn from the dissolution medium at predetermined time points and replaced with fresh dissolution medium. The collected samples were filtered using a 0.45 μm PTFE syringe filter, and then appropriately diluted with the dissolution medium. The concentration of orlistat dissolved in the medium was analyzed by the high performance liquid chromatography-ultraviolet (HPLC-UV) method. For comparison, raw orlistat and the commercial product, Xenical (Roche Pharmaceuticals, Nutley, NJ, USA), were also evaluated.

### 2.6. HPLC-UV Method for Orlistat Quantification

The HPLC-UV method was used to determine the orlistat concentration, using a Waters HPLC system consisting of a Waters 2690 Alliance analytical HPLC with auto-sampler and Waters 996 photodiode-array UV detector (Waters, Milford, MA, USA). The HPLC system was equipped with an XterraTM RP-18 column (150 × 4.6 mm, 5 µm, Waters, Milford, MA, USA). The mobile phase consisted of acetonitrile and water (95:5, v/v) and 0.1% (v/v) phosphoric acid. The flow rate was maintained at 1 mL/min and the UV-detection wavelength was set at 205 nm. The data acquisition and evaluation were performed with the Millennium 32 software (Waters, Milford, MA, USA).

### 2.7. In Vitro Oil Adsorption Test

The oil adsorption properties of various types of mesoporous silica were evaluated using a procedure described by Kumagai et al. [51]. First, 1 g of the sample was enclosed in a pack (70 mm × 80 mm) made of unwoven polypropylene fabric of 0.1 mm thickness. Then, it was dipped into olive oil for 30 min, without stirring. The bulk density of the used fabric was 0.20 g/cm^3^ and the diameter of the polypropylene fiber was 20 μm. The oil-adsorbed sample was, then, suspended for 30 min to allow the excess oil to drip away. The experiments were conducted at 23 ± 1 °C and at a relative humidity (RH) of 50% ± 5%. The weight of the oil adsorbed by the unwoven fabric, with and without the mesoporous silica, was measured and the difference between the two weights was noted to indicate the oil adsorption capacity.

### 2.8. In Vitro Lipase Inhibition Test

A lipase inhibition study was carried out, according to the procedure described by Dolenc et al., with slight modifications [52]. First, in order to find the orlistat concentration range where lipase inhibition is dependent on, orlistat aqueous solution in the range of 1 to 64 ug / mL was prepared by dissolving orlistat in DW containing 1% solutol HS15 at 37 °C using sonication. After confirming that the sample solution is clear, 2 mL of each solution at different orlistat concentration was filtered using a 0.45 μm PTFE syringe filter, then used for the determination of the lipase activity. To evaluate the lipase inhibition of prepared samples, the samples containing an equivalent of 10 mg of orlistat were placed in 900 mL of 1% SLS, maintained at 37 ± 0.1 °C and stirred at 75 rpm, using a USP II apparatus (VK7000, Vankel, Edison, NJ, USA). At predetermined time points, 2 mL of each sample was withdrawn and filtered using a 0.45 μm PTFE syringe filter. Filtered samples were used for the determination of the lipase activity. The lipase activity was analyzed by an enzymatic assay using quantification of the yellow colored *p*-nitrophenol (*p*-NP) product after hydrolysis of the chromogenic ester and *p*-NPP as the lipase substrate. Reagents for lipase inhibition tests were prepared just before the experiments. Solution I was prepared by mixing 41 mM Tris buffer (pH 8.4) with 1.8 mM sodium deoxycholate and 7.2 mM sodium taurodeoxycholate. Solution II consisted of 0.1 mM CaCl_2_ in 1.6 mM tartrate buffer (pH 4.0). The solution for enzymatic reaction was prepared as a mixture of 85% Solution I and 15% Solution II. For the preparation of the substrate solution, *p*-NPP was dissolved in acetonitrile in the stock solution to give a concentration of 10 mM. Next, the stock solution was diluted with solution for enzymatic reaction to give a final concentration of 0.25 mM. For the preparation of the lipase suspension, lipase powder from porcine pancreas was suspended in a pH 8.4 Tris buffer, at a concentration of 5 mg/mL. The suspension was centrifuged, and the supernatant was used for the enzymatic reaction. The enzyme assay experiments were conducted in a 96-well microplate. Seventy-eight microliters of the sample solution, 20 µL of lipase suspension, and 2 µL of the orlistat dissolution sample in 1% SLS of DW were added to the wells and then incubated at 37 °C. After pre-incubation for 20 min, absorbance at 405 nm was measured immediately after the addition of substrate *p*-NPP (100 µL), using Tecan Safire^®^ microplate reader (Tecan^®^ Trading AG, Männedorf, Switzerland). The absorbance was then measured at 60 s intervals for 60 min. The enzyme assay experiments were performed without orlistat and lipase as positive and negative controls, respectively. The activity and inhibition of lipase with orlistat were obtained from the following Equations (2) and (3):

(2) $$\%\;\mathrm{of}\;\mathrm{activity}\;\mathrm{of}\;\mathrm{lipase}=\frac{{\mathrm{slope}}_{\mathrm{avg}}\;\left(\mathrm{sample}\right)}{{\mathrm{slope}}_{\mathrm{avg}}\;\left(\mathrm{positive}\;\mathrm{control}\right)}\times 100$$

(3) % of inhibition=100−% of activity of lipase

### 2.9. In Vivo Animal Studies

The animal study protocol used was in compliance with the institutional guidelines for the care and use of laboratory animals and was approved by the ethics committee of Kyungsung University (No. 18-023A, Approved Date: 14 October 2018). The grouped rats were housed in a cage and maintained in a 12 h light/dark cycle at room temperature (25 °C) and an RH of 55% ± 10%. General and environmental conditions were strictly monitored. 

#### 2.9.1. Serum Triglyceride Levels in Sprague Dawley (SD) Rats after Administration of Orlistat Formulation

To evaluate the in vivo pharmacological efficacy of the orlistat formulation, serum TG levels were measured after administration of orlistat. Male SD rats (7 weeks old), weighing between 200 and 220 g, were obtained from Samtaco Bio Korea Inc. (Osan, Korea). All rats had free access to tap water and a normal pelleted diet. The rats were divided into eight groups of six animals each and were fasted overnight before the experiment. Each experimental group was orally administered 1 mL of olive oil, followed by 0.25% w/v HPMC aqueous suspensions containing formulations equivalent to 2.5 mg/kg body weight as orlistat. Raw orlistat, the commercial product (Xenical^TM^, Roche Pharmaceuticals, Nutley, NJ, USA), and five SCMA processed orlistat formulations with a 20% drug loading ratio were administered to the experimental groups. For comparison, the control group was orally administered 1 mL of olive oil without the drug. Blood samples were taken from the tail vein of the rats at 0 (baseline), 0.5, 1, 2, 3, 4, 5, 6, 8, and 12 h after administration of the formulation/placebo. Blood samples were kept on ice (+4 °C) until centrifugation at 4000 rpm at 15 °C for 15 min. Serum was transferred to individual Eppendorf tubes and stored at −20 °C until analysis. The serum TG concentration was determined using a commercially available assay kit, Cleantech TG-S (Asan Pharmacy Ltd., Hwaseong, Korea), based on the measurement of glycerol enzymatically hydrolyzed from TGs. The increase in the serum TG levels (ΔTG) after the oral administration of the orlistat formulation was calculated by subtracting the fasting/baseline levels from the serum TG levels.

#### 2.9.2. Data Analysis

The maximal ΔTG (ΔTG_max_) was obtained directly from the ΔTG-time curve. The area under the curve (AUC) was calculated from the ΔTG-time curve using WinNonlin 2.1 (Pharsight Corporation, Mountain View, CA, USA).

#### 2.9.3. Fat Excretion via Feces in ICR Mice

Male ICR mice (4 weeks old), weighing between 15 and 20 g, were obtained from Samtaco Bio Korea Inc. (Osan, Korea). Animals were given free access to tap water and a high fat diet, D12492 (Research Diets, Inc., New Brunswick, NJ, USA), which provided 60% of the total calories as fat. The mice were divided into four groups of six animals each. Raw orlistat, the commercial product (Xenical^TM^, Roche Pharmaceuticals, Nutley, NJ, USA), and a selected orlistat formulation (with a 20% drug loading ratio) with largest AUC in TG-time curve were used in three experimental groups while the fourth group was the control group. Each experimental group was orally administered 0.25% w/v HPMC aqueous suspension, containing orlistat equivalent to 2.5 mg/kg body weight, at 9 am and 6 pm for 5 days. For comparison, the positive control group was orally administered 1 mL of DW. After 5 days, the feces were collected and then freeze-dried. The fat in the dried feces was extracted using a slightly modified method reported by Smedes et al. [53]. Briefly, 2.5 g of dried feces samples were placed in 50 mL conical tubes. Eight milliliters of propan-2-ol and 5 mL of cyclohexane were added to the tube and the mixture was vortexed for 2 min. Five milliliters of cyclohexane was added and the mixture was shaken vigorously for 2 min. Next, 11 mL of DW was added and the mixture was vortexed for 2 min. The phases were separated by centrifugation for 10 min at 2000 rpm. The supernatants were transferred to a pear-shaped flask. A second extraction was performed with 20 mL of 10% (v/v) propan-2-ol in cyclohexane by vortexing for 2 min. After centrifugation, the cyclohexane phase was added to the first extract. Evaporation was done with a rotary evaporator (N-1110, EYELA, Bohemia, NY, USA), and the residue was further dried at 80 °C for 1 h in an oven. The residues were weighed and used to calculate fat content in feces (mg/g), based on the amount of fat remaining per unit weight of dried feces.

#### 2.9.4. Oily Spotting Test

Male SD rats (7 weeks old), weighing between 200 and 220 g, were obtained from Samtaco Bio Korea Inc. (Korea). All rats had free access to tap water and a normal pelleted diet. The rats were divided into eight groups of twenty-six animals each and were fasted overnight before the experiment. Raw orlistat, commercial product (XenicalTM, Roche Pharmaceuticals, Nutley, NJ, USA), and five SCMA-processed orlistat formulations (with a 20% drug loading ratio) were used for the seven experimental groups while the eighth group was the control group. Each experimental group was orally administered 0.5 mL of olive oil, followed by 0.25% w/v HPMC aqueous suspension, containing orlistat equivalent to 5 mg/kg body weight. For comparison, the control group was orally administered 0.5 mL of olive oil. This experiment was based on the experimental observation that when rats are orally administered olive oil, they spread the excreted free oil over their fur while grooming. The observation of free oil was conducted at 6 h after administration, and the number of rats having oily spotting was counted for each group.

### 2.10. Statistical Analysis

Statistical analysis was performed by the independent T-test or a one-way analysis of variance (ANOVA), followed by Student–Newman–Keuls (SNK) tests using the SPSS 12.0 software (IBM SPSS, Chicago, IL, USA).

## 3. Results and Discussions

### 3.1. Physicochemical Characterization of Orlistat-Loaded Mesoporous Silica

#### 3.1.1. Morphology of Mesoporous Silica before and after Orlistat Adsorption by SCMA

**Figure 2:**
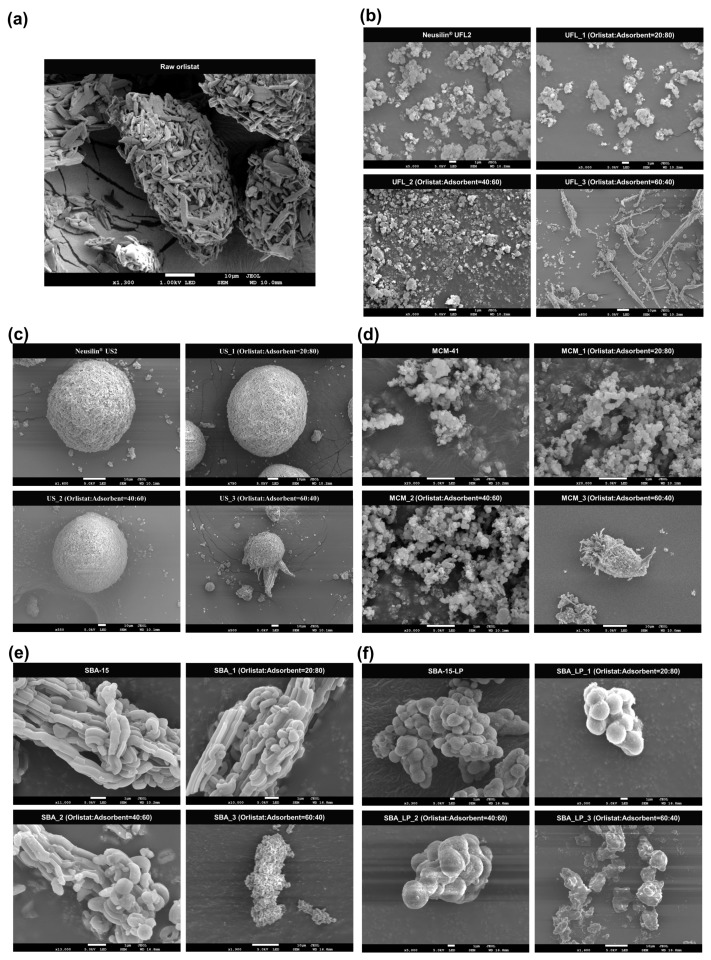
SEM images of particles with various raw mesoporous silica and orlistat adsorbed on them at three different drug loading ratio by SCMA process: (**a**) raw orlistat, (**b**) Neusilin^®^ UFL2; (**c**) Neusilin^®^ US2; (**d**) MCM-41; (**e**) SBA-15; and (**f**) SBA-15_LP.

SEM photographs of various mesoporous silica before and after orlistat adsorption by SCMA are shown in Figure 2. Raw Neusilin^®^ UFL2 and MCM-41 showed secondary agglomeration of particles, with a large surface area. Neusilin^®^ US2 consisted of small spherical particles with a size of a few microns. The morphology of SBA-15 was fiber-like and the particles were several tens of micrometers in length, composed of basic rod-like structures, similar to the morphology observed in previous studies [54,55,56,57]. In comparison to SBA-15, the SBA-15-LP particles appeared as secondary agglomerates composed of spherical particles [49].

The SEM image analyses revealed no distinct difference in the surface morphologies between pure mesoporous silica and orlistat-loaded mesoporous silica below a 40% drug loading ratio in the formulation. However, particles with crystal growth were observed on the surface or outside of mesoporous silica at 60% drug loading ratio. This indicated that the pore volume of mesoporous silica was insufficient for hosting the extra orlistat molecules at a 60% drug loading ratio; hence, the residual orlistat was observed as solid crystals outside the adsorbent pore [58,59,60]. A more detailed explanation regarding this observation will be discussed later with respect to total pore volume and pore diameter.

#### 3.1.2. Specific Surface Area, Total Pore Volume, and Pore Diameter of Various Mesoporous Silica before and after Orlistat Adsorption by SCMA

The specific surface area, total pore volume, and pore diameter of various mesoporous silica are summarized in Table 1. The large specific surface area and total pore volume of all mesoporous silica indicate that they are desirable core materials for the adsorption of a relatively high drug loading ratio. As reported by Jana et al., SBA-15-LP with a larger pore diameter than SBA-15 was successfully synthesized at a molar ratio of SiO_2_:1,2,3-TMB = 1:2. 

**Figure 3:**
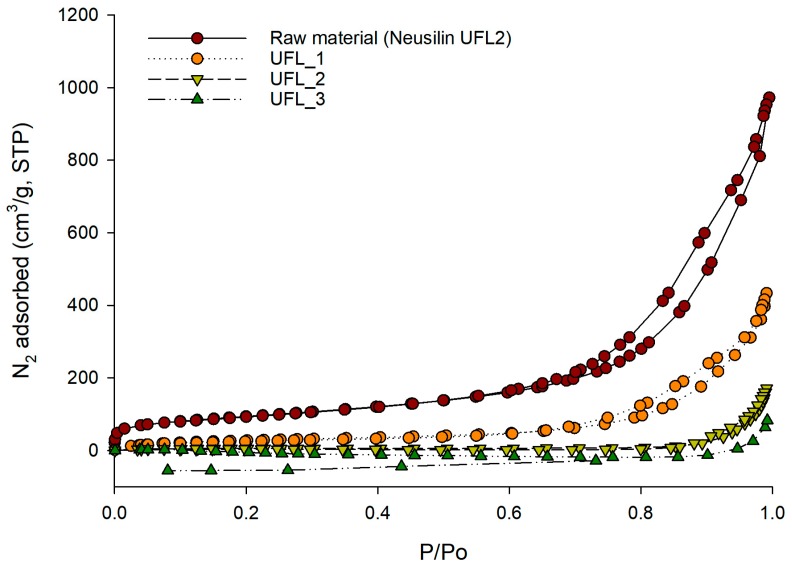
Nitrogen adsorption/desorption isotherms for raw Neusilin^®^UFL2 and orlistat-loaded Neusilin^®^UFL2 at three different drug loading ratio.

For the orlistat-loaded mesoporous silica prepared by the SCMA process, both the specific surface area and total pore volume of various mesoporous silica decreased with an increase in orlistat loading mass ratio to mesoporous silica. This indicated that the pores of mesoporous silica were filled with orlistat, and both the specific surface area and total pore volume of the drug-loaded mesoporous silica decreased as the number of pores filled with orlistat increased (Table 1). This explanation was also confirmed by the nitrogen adsorption/desorption isotherms of the selected orlistat-loaded Neusilin^®^ UFL2 samples. As shown in Figure 3, the pores of Neusilin^®^ UFL2 were filled and the void volume decreased as the mass ratio of orlistat increased; hence, the adsorption of nitrogen decreased.

#### 3.1.3. DSC and PXRD Analyses

**Figure 4:**
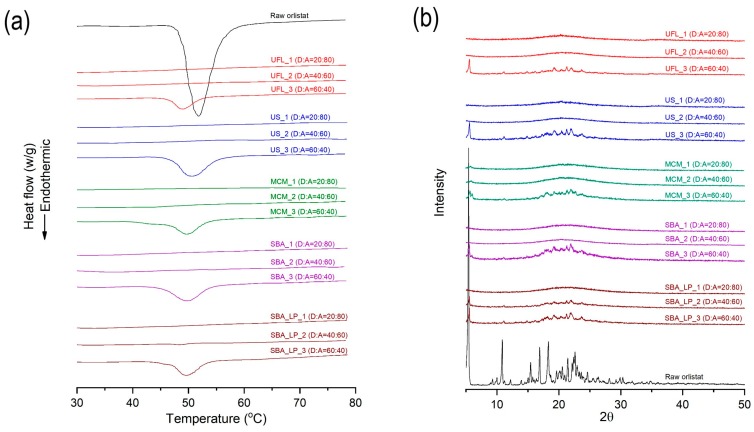
DSC thermograms (**a**) and PXRD patterns (**b**) of raw orlistat and orlistat-loaded mesoporous silica at 20%, 40%, and 60% drug loading ratio. D:A is the mass ratio of drug to adsorbent.

The DSC thermograms and PXRD patterns for raw orlistat and various types of mesoporous silica, before and after orlistat adsorption by the SCMA process, are presented in Figure 4. Raw orlistat was characterized as a crystalline form by a single melting endothermic peak at 51.6 °C (ΔH = 76.37 J/g) in the DSC thermogram and typical diffraction peaks of the crystalline structure of orlistat were observed at 5.4°, 10.8°, 15.5°, 16,8°, 18.1°, 21.4°, and 22.6° in the PXRD pattern, which corresponded to the results obtained in an earlier study about pure crystalline orlistat [61]. For all the types of pure mesoporous silica used, neither an endothermic peak in DSC analysis nor diffraction peaks in PXRD patterns were detected because of the amorphous structure. As shown in Table 1 and Figure 4, it was observed that the crystallinity decreased with an increase in the adsorbent ratio. When the mass ratio of orlistat:adsorbent was 60:40, both the endothermic peak of DSC and the diffraction peaks of the PXRD patterns were detected. As shown in Table 1, formulations with 60% drug loading exceeded the theoretical maximum loading percentage of all mesoporous silica types used. Therefore, it could be suggested that for formulations having a loading percentage above the theoretical maximum, the pore volume of the adsorbent was insufficient for hosting the extra orlistat molecules; hence, the extra orlistat was crystallized on the external surface of the adsorbent instead of inside the pores. These results were confirmed by the SEM images (Figure 2) that show the presence of particles with crystal growth on the surface or outside the mesoporous silica at 60% drug loading ratio. Interestingly, the results of DSC and PXRD showed that in the orlistat-loaded SBA-15_LP, a small portion of the orlistat was in a crystalline state, even at 40% drug loading, which was lower than the theoretical maximum loading percentage 47.9%. As shown in the SEM images (Figure 2), there were no remarkable differences in the surface morphologies between SBA-15_LP before and after orlistat adsorption 40% drug loading. Such exceptional observation can be explained based on the results reported by Sliwinska-Bartkowiak et al. [62]. They found that the confined fluid molecules solidify into a single crystalline structure in mesoporous silica, with pore diameters greater than 20 times the size of the fluid molecule. For average pore sizes between 20 and 15 times the size of the fluid molecule, a portion of the confined fluid molecules solidifies into a frustrated crystal structure, while the rest form an amorphous region. For pore sizes smaller than 15 times the size of the fluid molecule, even partial crystallization does not occur. The calculated molecular size of orlistat was 1.6–2.1 nm, which was estimated by molecular modeling using Chem-Bio 3D Ultra version 12.0 (CambridgeSoft, Cambridge, MA, USA), following energy minimization with molecular mechanics (MM2) (Figure 1). The pore sizes of the used adsorbents, except for SBA-15_LP, were smaller than 15 times the size of the orlistat molecule. 

In contrast, the pore size of SBA-15_LP was 37.21 nm, which is about 18–23 times larger than the molecular size of orlistat. Hence, it was suggested that a partial crystallization of orlistat occurred within the pores of SBA-15_LP (Figure 4).

### 3.2. In Vitro Dissolution

Powder dissolution profiles for raw orlistat, the commercial product, and orlistat-loaded mesoporous silica samples with different mass ratios are shown in Figure S1 (Supplementary Material) and the calculated dissolution efficiency values at 60 min (DE_60_) are presented in Table 1. The dissolution of orlistat from drug-adsorbed mesoporous silica was higher than that for raw orlistat and the commercial product. For all types of mesoporous silica used, drug release was markedly enhanced in formulations with an orlistat loading ratio below 40% compared to 60%. This could be resulted from both the increased surface area of orlistat after adsorption onto mesoporous silica and the decrease in crystallinity with the increase in the amorphous portion at orlistat loading below 40% [30]. Amorphous systems do not require an input of energy for the breakage of the crystal lattice and, thus, have a solubility advantage compared to the solid crystalline forms. Therefore, lower crystallinity results in a higher dissolution rate [63].

A comparative study of the different mesoporous silica revealed that the DE_60_ of the prepared orlistat formulations with 20% and 40% drug loading ratios decreased in the following order: Neusilin^®^UFL2 > SBA-15 > Neusilin^®^US2 > SBA-15_LP > MCM-41. This indicated that not only the crystallinity of the drug but also the pore diameter of the adsorbent significantly influenced the dissolution rate because the pore size determined the diffusion rate of the drug from the pore channel [27,64]. Since the pore diameter of Neusilin^®^UFL2 was larger than that of other adsorbents, a faster diffusion rate of orlistat from the pores of Neusilin^®^UFL2 might result in a faster dissolution rate. On the other hand, both MCM_1 and MCM_2, prepared using MCM-41 with the smallest pore diameter, showed the lowest dissolution rate compared to other formulations prepared with the same orlistat loading ratio. In contrast, the pore size of SBA-15_LP was larger than that of SBA-15, but the DE_60_ of SBA-15_LP was lower than SBA-15 at 20 and 40% drug loading ratio. This can be explained by the slower dissolution kinetics of crystalline orlistat compared to its amorphous form [61]. Although the large pore size of SBA-15-LP facilitated the diffusion of the adsorbed drug from the internal pores of SBA-15_LP to the dissolution medium, a small portion of crystalline orlistat that can be detected or is below the lower limit of detection dominated the overall dissolution profile; hence, it exhibited a slower dissolution rate. Furthermore, it was revealed that the particle size of mesoporous silica also influenced the dissolution rate. Although the pore size of Neusilin^®^UFL2 was similar to that of Neusilin^®^US2, the DE_60_ of Neusilin^®^UFL2 was much higher than that of Neusilin^®^US2 due to the former’s smaller particle size (Figure 2).

### 3.3. In Vitro Lipase Inhibition

**Figure 5:**
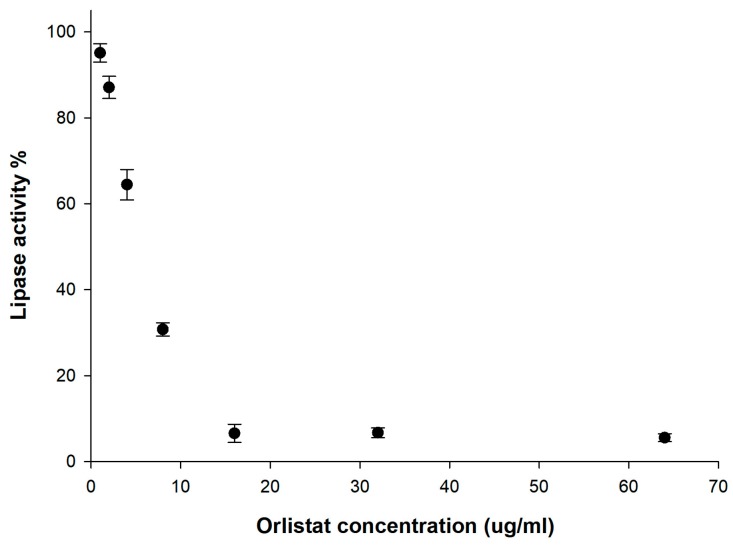
Influence of orlistat concentration on lipase activity.

Orlistat inhibits both gastric and pancreatic lipases as well as carboxyl esterase by reacting with the catalytic serine residue of these enzymes [65]. To determine the biological potency of the orlistat formulations prepared with various mesoporous silica, an in vitro lipase inhibition study was carried out. As shown in Figure 5, the orlistat inhibited the lipase activity in a dose-dependent manner as follows: 1 µg/mL (4.88% ± 2.13%), 2 µg/mL (12.91% ± 2.58%), 4 µg/mL (35.54% ± 3.55%), and 8 µg/mL (69.26% ± 1.54%). When 16 µg/mL orlistat was added, a total inhibition of lipase activity was observed.

**Figure 6:**
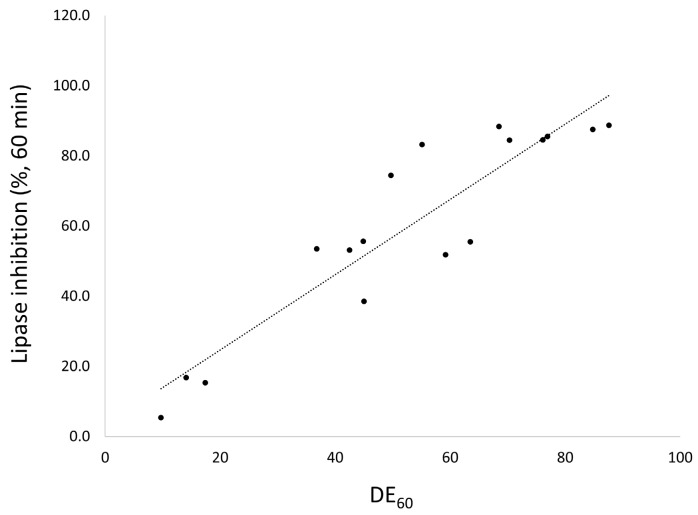
The relationship between DE_60_ and lipase inhibition % at 60 min.

The lipase activity profiles for raw orlistat, the commercial product and the prepared formulations with different drug ratios using the SCMA method are shown in Figure S2 (Supplementary Material) and the % inhibition of lipase in samples collected at 60 min, are presented in Table 1. A comparative study of the different mesoporous silica revealed that the percentage of lipase inhibition for the prepared orlistat formulation at 40% drug loading ratio decreased in the following order: Neusilin^®^UFL2 ≥ Neusilin^®^US2 > SBA-15 > MCM-41 > SBA-15_LP. The lipase activities of orlistat-loaded mesoporous silica were significantly decreased compared to both raw orlistat and the commercial product. In addition, above 80% of lipase activity was inhibited within 10 min for both UFL_1 and UFL_2, while raw orlistat and the commercial product showed 1.5% and 6.7% inhibition, respectively (Figure S2). From these results, it was revealed that the percentage of lipase inhibition increased with increasing DE (Figure 6). Consequently, it can be suggested that the orlistat in orlistat-loaded Neusilin^®^ UFL2 rapidly achieved saturation, thus inhibiting the lipase activity more efficiently due to a higher dissolution rate than other formulations [52].

### 3.4. In Vitro Oil Adsorption Capacity of Mesoporous Silica

**Figure 7:**
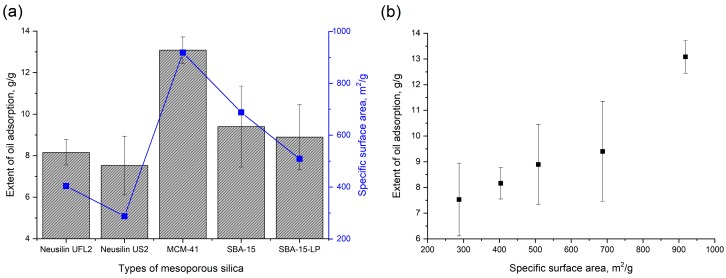
(**a**) Oil adsorption capacity of mesoporous silica and (**b**) relationship with specific surface area.

To predict the undigested lipid adsorption capacity of various mesoporous silica after the in vivo release of the adsorbed drug, an in vitro oil adsorption test was conducted. The results showed that the oil adsorption capacity increased with an increase in the specific surface area of mesoporous silica, and MCM-41 with the largest surface area has the highest oil adsorption capacity (Figure 7). A similar trend was reported previously by Zhao et al. [66]. 

**Figure 8:**
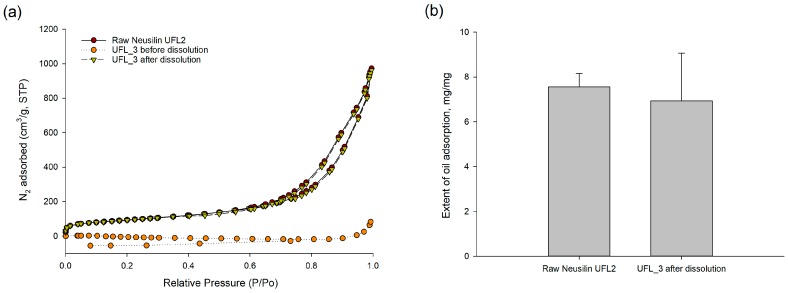
(**a**) Oil adsorption capacity of orlistat-loaded Neusilin^®^UFL2 (UFL_3, 60% drug loading ratio) after dissolution test (**b**) and nitrogen adsorption isotherms both before and after dissolution test.

Furthermore, the oil adsorption capacity of mesoporous silica after dissolution of orlistat from the prepared formulation was also evaluated. The nitrogen adsorption isotherms and oil adsorption capacity of orlistat-loaded Neusilin^®^UFL2 after the dissolution study are shown in Figure 8a. After the drug adsorbed onto the silica pores was completely dissolved, total pore volume and specific surface area were restored to original state of raw Neusilin^®^UFL2 before drug adsorption. Hence, there was no significant difference in oil adsorption capacity between before orlistat adsorption and after the dissolution test of orlistat-loaded Neusilin^®^UFL2 (Figure 8b). These results indicate that the regular mesoporous structure of the host material was preserved during the loading procedure and the dissolution test. Thus, it was hypothesized that the desirable oil adsorption capacity of mesoporous silica material could help reduce the side effects of orlistat, such as fatty/oily stool and oily spotting, by adsorption of undigested oil or fat by empty mesoporous silica after the release of orlistat in the gastrointestinal tract.

### 3.5. In Vivo Study of Orlistat-Loaded Mesoporous Silica

#### 3.5.1. Serum TG Level

**Figure 9:**
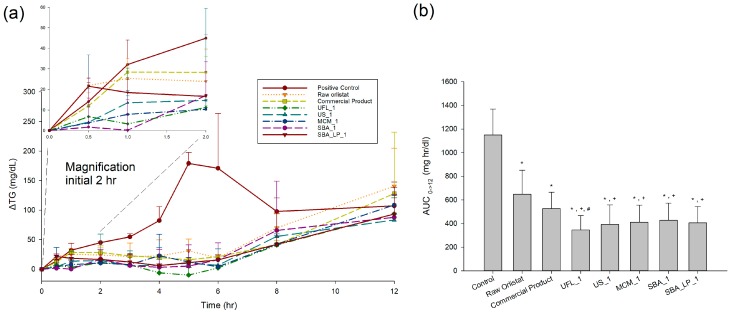
(**a**) ΔTG vs. time profiles and (**b**) AUC_0→12_ of ΔTG in SD-rats, after oral administration 1 mL olive oil, followed by administration of raw orlistat, the commercial product, and SCMA processed orlistat formulations (n = 6). *: *p* < 0.05, vs. control; ^+^: *p* < 0.05, vs. raw orlistat; ^#^: *p* < 0.05, vs. commercial product.

Figure 9a shows the time profiles of ΔTG levels after oral administration of 1 mL olive oil, followed by administration of orlistat-adsorbed mesoporous silica formulations, with a 20% drug loading ratio. ΔTG is the difference in the serum TG levels from the fasting/baseline levels. For comparison, raw orlistat and the commercial product were also evaluated. The pharmacokinetic (PK) parameters, calculated from ΔTG vs. time profiles, are presented in Table 2. In case of the control (without orlistat), the area under the ΔTG-time curve (AUC_0→12_) and ΔTG_max_ were 1149.2 ± 219.4 mg·h/dL and 179 ± 18.9 mg/dL, respectively. The serum TG level was dramatically decreased for both groups of raw orlistat and the commercial product when compared to the control group. However, there was no significant difference in AUC_0→12_ and ΔTG_max_ between raw orlistat and the commercial product. In the administered groups of raw orlistat and commercial products, TG increased up to 1 h after administration with negligible difference from the control group, and then the rate of increase was decreased. On the other hand, most of the orlistat-loaded mesoporous silica samples inhibited the increase in TG more quickly. In addition, the ANOVA showed that there were significant differences in PK parameters between commercial product and orlistat-loaded mesoporous silica samples prepared by SCMA method (*p* < 0.05), which were ranked by the SNK test in order of increasing AUC_0→12_ as follows: UFL_1 < US_1 < SBA_LP_1 < MCM < SBA_1 < commercial product < raw orlistat < control (Figure 9b). When compared to raw orlistat and commercial products, the highest AUC obtained with UFL_1 among the experimental groups and the higher AUC values observed for all orlistat-loaded mesoporous silica samples coincide well with the results of the in vitro dissolution and lipase inhibition studies. These results suggest that the amorphous nature and increased surface area of orlistat in the developed formulation allow for greater solubility, thereby increasing the in vivo dissolution rate and drug concentration available for inhibition of lipase in the gastrointestinal tract [52,67,68,69].

#### 3.5.2. Fat Excretion via Feces

**Figure 10:**
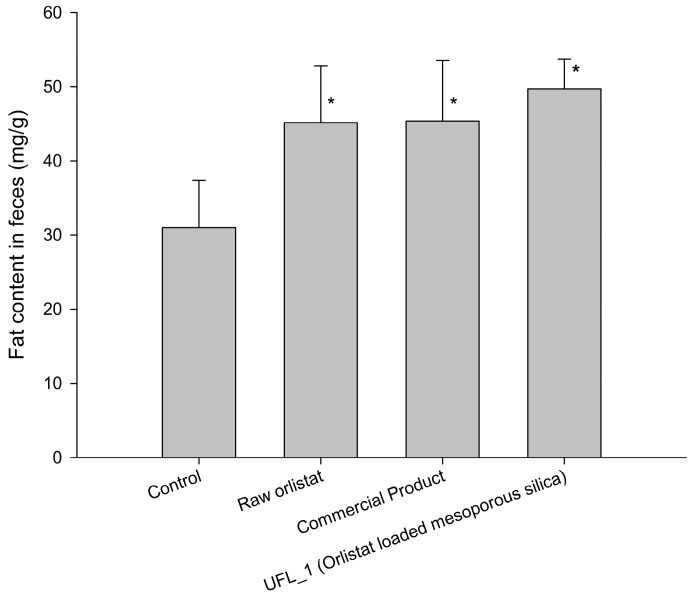
Effects of raw orlistat, the commercial product, and UFL_1 on fat excretion in feces of mice fed a high-fat diet for 5 days. *: *p* < 0.05, vs. control.

Figure 10 shows the effects of raw orlistat, the commercial product, and UFL_1 formulation (orlistat: Neusilin^®^UFL2 = 20:80, w/w) on fat excretion in feces of mice on day 5 after feeding a high-fat diet. The fat content in the feces of mice fed with a high fat diet without orlistat (control group) was 30.97 ± 6.43 mg/g. After administration of raw orlistat, the commercial product, and UFL_1, the fat content in the feces significantly increased to 45.15 ± 7.64, 45.37 ± 8.18, and 49.71 ± 4.01 mg/g, respectively, when compared to the control group (*p* < 0.05). This implied that orlistat inhibited the activity of lipase. Hence, TGs from the food were not hydrolyzed into absorbable free fatty acids and were excreted as undigested free oil via feces. However, there was no significant difference in fat content in the feces between the groups administered UFL_1, raw orlistat, and the commercial product.

#### 3.5.3. Oily Spotting Number

**Figure 11:**
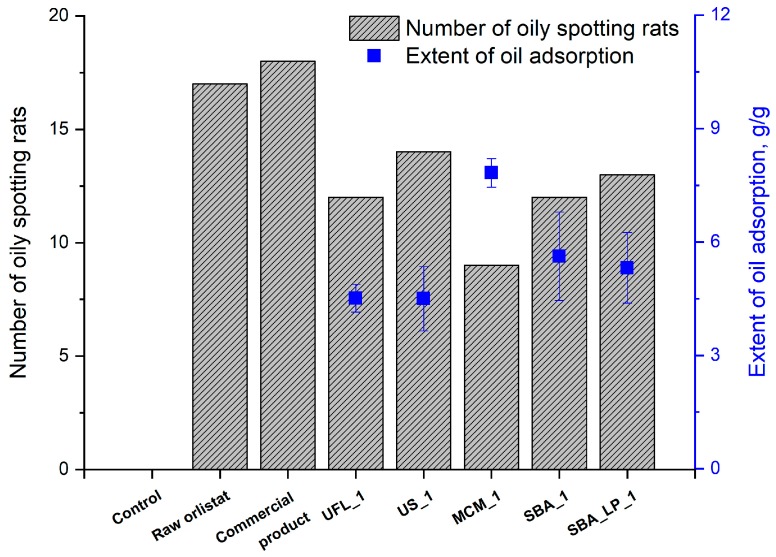
Number of rats showing oily spotting after administration of raw orlistat, the commercial product (Xenical^®^), and orlistat-loaded mesoporous silica (n = 26).

To assess the impact of orlistat formulation with mesoporous silica in reducing the side effects of orlistat, the number of oily spots in rats was counted after administration of 0.5 mL olive oil, followed by administration of orlistat formulations with a 20% drug loading ratio. As shown in Figure 11, the number oily spots decreased in the following order: commercial product > raw > US_1 > SBA_LP_1 > UFL_1 = SBA_1 > MCM_1. As expected, the number of rats with oily spotting in the control group without orlistat was much smaller than the number in the experimental groups administered orlistat because of the lipase inhibition by orlistat. Interestingly, the number of rats with oily spotting decreased in all groups administered SCMA-processed orlistat formulations with mesoporous silica compared to groups administered raw orlistat or the commercial product. This result was probably obtained because all types of mesoporous silica used as adsorbents in this study could adsorb undigested oil in the intestinal tract, as intended, via empty mesoporous silica after releasing the adsorbed orlistat. In particular, the number of rats with oily spotting was lowest in the MCM_1 group. This observation is in agreement with the nature of MCM-41, which has the highest in vitro oil adsorption capacity due to the fact that it has the largest surface area among the various types of mesoporous silica, as shown in Figure 7.

## 4. Conclusions

In this study, orlistat was loaded onto five types of mesoporous silica with various surface areas, pore volumes, and diameters by the SCMA method using SC-CO_2_. The results showed that orlistat was successfully loaded onto mesoporous silica in an amorphous state in all types of mesoporous silica at a drug loading ratio of 20%. Further, it was observed that the surface area, total pore volume, and pore diameter of mesoporous silica played major roles in determining the crystallinity, dissolution rate, inhibition of lipase activity, and oil adsorption capacity. The pore volume of silica and the relative ratio of the pore diameter of silica compared to the drug molecular size had significant effects on the loadable drug amount inside pores and crystallinity. The low crystallinity of orlistat and the large pore diameter and small particle size of the used mesoporous silica contributed to faster dissolution. The serum TG levels decreased significantly in rats that were administered orlistat-loaded mesoporous silica compared to those that were administered raw orlistat or the commercial product. This was attributed to the amorphous nature and increased surface area of orlistat after loading onto mesoporous silica. The UFL_1 formulation (orlistat-loaded Neusilin^®^ UFL2 with a 20% loading ratio) showed the highest in vivo pharmacological efficacy with the lowest AUC of serum TG level. This formulation also showed the highest in vitro dissolution rate and inhibition of lipase activity. Fat excretion via feces in mice that were administered UFL_1 was significantly decreased compared to the control group, but not in comparison with groups administered raw orlistat and the commercial product. Furthermore, the oily spotting study revealed that the empty mesoporous silica, after releasing the adsorbed orlistat, could successfully adsorb the undigested free oil. Therefore, it was concluded that the preparation of orlistat-loaded mesoporous silica, using the SCMA process with SC-CO_2_, could be used as a promising strategy to improve the solubility of orlistat and overcome the side effects of the commercial product.

## Figures and Tables

**Table 1:** Experimental results of raw materials, the commercial product, various mesoporous silica, and orlistat-loaded mesoporous silica.

Formulation		D:A (w/w)^1^	Specific Surface Area (m^2^/g)	Total Pore Volume (cm^3^/g)	Pore Diameter (nm)	%DL_max_theo_^2^	RC^3^ (%)	Solid States^4^	DE_60_^5^	Lipase Inhibition (%)^6^
Pure adsorbent	Neusilin^®^UFL2	0:100	403.93	1.4137	17.01	58.1	-	Amorphous	-	-
	Neusilin^®^US2	0:100	287.65	0.9568	16.45	48.4	-	Amorphous	-	-
	MCM_41	0:100	918.27	1.0333	4.09	50.3	-	Amorphous	-	-
	SBA_15	0:100	687.65	0.9617	5.59	48.5	-	Amorphous	-	-
	SBA_15_LP	0:100	508.62	0.9385	37.21	47.9	-	Amorphous	-	-
SCMA processed with orlistat	UFL_1	20:80	105.21	0.6491	-	-	0	Amorphous	87.6 ± 0.6	88.8 ± 3.4
	UFL_2	40:60	19.91	0.2511	-	-	0	Amorphous	84.8 ± 3.1	87.5 ± 2.5
	UFL_3	60:40	6.42	0.0531	-	-	32.0	Amorphous+Crystal	55.1 ± 1.0	83.2 ± 4.8
	US_1	20:80	149.44	0.0690	-	-	0	Amorphous	70.3 ± 3.3	84.5 ± 1.7
	US_2	40:60	49.70	0.0229	-	-	0	Amorphous	68.5 ± 1.2	88.4 ± 0.6
	US_3	60:40	24.69	0.0111	-	-	52.0	Amorphous+Crystal	49.7 ± 0.7	74.5 ± 2.6
	MCM_1	20:80	759.93	0.3506	-	-	0	Amorphous	44.9 ± 1.3	55.7 ± 2.5
	MCM_2	40:60	34.35	0.0162	-	-	0	Amorphous	42.5 ± 1.3	53.2 ± 2.3
	MCM_3	60:40	7.12	0.0030	-	-	41.4	Amorphous+Crystal	36.8 ± 1.0	53.5 ± 2.4
	SBA_1	20:80	281.98	0.1358	-	-	0	Amorphous	76.9 ± 0.4	85.6 ± 5.5
	SBA_2	40:60	45.51	0.0209	-	-	0	Amorphous	76.1 ± 1.9	84.6 ± 1.4
	SBA_3	60:40	20.724	0.0097	-	-	50.3	Amorphous+Crystal	45.0 ± 1.0	38.5 ± 9.0
	SBA_LP_1	20:80	127.92	0.0651	-	-	0	Amorphous	63.5 ± 1.4	55.5 ± 0.0
	SBA_LP_2	40:60	22	0.0043	-	-	4.1	Amorphous+Crystal	59.2 ± 1.0	51.9 ± 1.4
	SBA_LP_3	60:40	6.57	0.0036	-	-	68.8	Amorphous+Crystal	17.4 ± 0.3	15.3 ± 1.2
Commercial product		-	-	-	-	-	99.2	Crystal	14.1 ± 1.3	16.8 ± 1.9
Raw orlistat		100:0	-	-	-	-	100	Crystal	9.7 ± 1.1	5.4 ± 0.7

^1^ Mass ratio of drug to adsorbent, ^2^ Theoretical maximum loading percentage (%) in the prepared formulation calculated by %DL_max_theo_ = [M_ads_theo_/(1 + M_ads_theo_)] × 100, where M_ads_theo_ is the mass of drug (g) corresponding to the pore volume of 1 g of adsorbent (In other words, the drug mass (g) that can theoretically be loaded inside the pore of 1 g adsorbent), which is calculated by multiplying total pore volume (cm^3^/g) of adsorbent by true density of orlistat (0.98 g/cm^3^), ^3^ Relative crystallinity of the sample obtained by DSC measurement in comparison with raw orlistat, ^4^ Solid state of orlistat determined by DSC and PXRD results, ^5^ Dissolution efficiency at 60 min of dissolution test, ^6^ Lipase inhibition % at 60 min.

**Table 2:** Pharmacokinetic (PK) parameters of ΔTG in SD-rats, after oral administration 1 mL olive oil, followed by administration of raw orlistat, the commercial product, and SCMA processed orlistat formulations (n = 6).

Formulation	PK Parameter of ΔTG		Relative PK Parameter (%)					
	ΔTG_max_ (mg/dL)	AUC_0→12 h_ (mg·hr/dL)	Compare to Control		Compare to Raw Orlistat		Compare to Commercial Product	
			ΔTG%^1^	AUC%^2^	ΔTG%^3^	AUC%^4^	ΔTG%^5^	AUC%^6^
Control	179.0 ± 18.9	1149.2 ± 219.4	-	-	-	-	-	-
Raw orlistat	140.7 ± 64.1	648.0 ± 203.2	78.6	56.4	-	-	-	-
Commercial product	128.0 ± 54.0	526.4 ± 138.9	71.5	45.8	91.0	81.2	-	-
UFL_1	93.5 ± 27.5	345.9 ± 120.3	52.2	30.1	66.5	53.4	73.0	65.7
US_1	83.3 ± 10.1	391.9 ± 164.9	46.5	34.1	59.2	60.5	65.1	74.4
MCM_1	108.7 ± 18.3	410.2 ± 145.2	60.7	35.7	77.3	63.3	84.9	77.9
SBA_1	87.8 ± 37.5	427.7 ± 144.8	49.1	37.2	62.4	66.0	68.6	81.3
SBA_LP_1	93.4 ± 45.2	406.9 ± 136.5	52.2	35.4	66.4	62.8	73.0	77.3

^1^ (ΔTG_max_ of sample/ ΔTG_max_ of control) × 100, ^2^ (AUC_0**→**12 h_ of sample/ AUC_0**→**12 h_ of control) × 100, ^3^ (ΔTG_max_ of sample/ ΔTG_max_ of raw orlistat) × 100, ^4^ (AUC_0**→**12 h_ of sample/ AUC_0**→**12 h_ of raw orlistat) × 100, ^5^ (ΔTG_max_ of sample/ ΔTG_max_ of commercial product) × 100, ^6^ (AUC_0**→**12 h_ of sample/ AUC_0**→**12 h_ of commercial product) × 100.

## Supplementary Materials

The following are available online at https://www.mdpi.com/1999-4923/12/4/333/s1, Figure S1: Powder dissolution profiles of orlistat loaded onto mesoporous silica at three different drug loading ratio prepared using SCMA process: (a) Neusilin^®^UFL2; (b) Neusilin^®^US2; (c) MCM-41; (d) SBA-15; and (e) SBA-15_LP. Figure S2: Inhibition of lipase by orlistat loaded onto mesoporous silica at three different drug loading ratio prepared using SCMA process: (a) Neusilin^®^UFL2; (b) Neusilin^®^US2; (c) MCM-41; (d) SBA-15; and (e) SBA-15_LP.
