# Supplementary material for: Pharmaceutical Characterization and In Vivo Evaluation of Orlistat Formulations Prepared by the Supercritical Melt-Adsorption Method Using Carbon Dioxide: Effects of Mesoporous Silica Type
Source: Pharmaceutics. 2020 Apr 8;12(4):333. doi: 10.3390/pharmaceutics12040333 (PMC7238058; doi:10.3390/pharmaceutics12040333)
Supplement: Supplementary file 1 [file pharmaceutics-12-00333-s001.pdf]

pharmaceutics

Supplementary Materials: Pharmaceutical Characterization and in Vivo Evaluation of Orlistat Formulations Prepared by the Supercritical Melt-Adsorption Method Using Carbon Dioxide: Effects of Mesoporous Silica Type

Heejun Park, Kwang-Ho Cha, Seung Hyeon Hong, Sharif Md Abuzar, Seungyeol Lee, Eun-Sol Ha, Jeong-Soo Kim, In-Hwan Baek, Min-Soo Kim and Sung-Joo Hwang

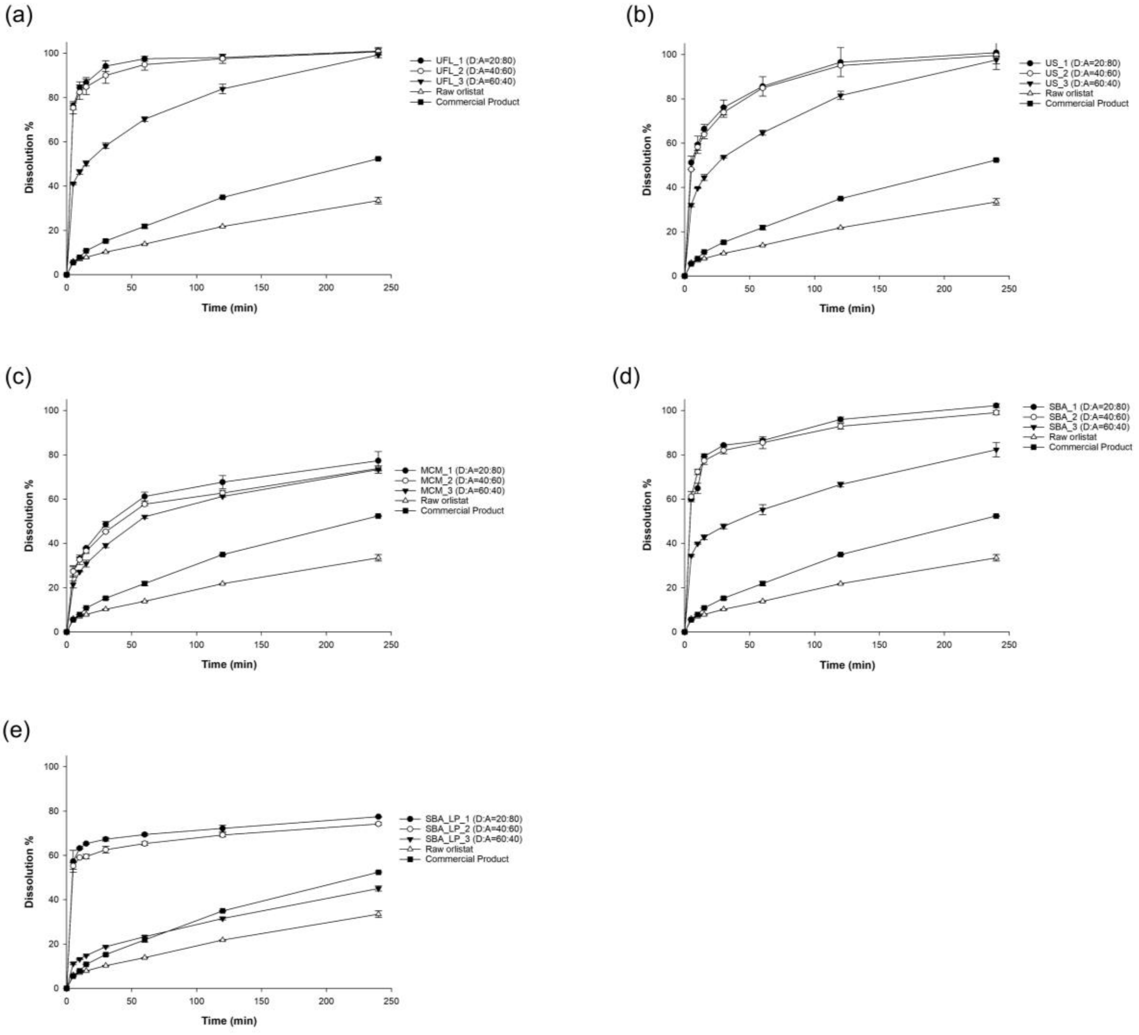

Figure S1. Powder dissolution profiles of orlistat loaded onto mesoporous silica at three different drug loading ratio prepared using SCMA process: (**a**) Neusilin®UFL2; (**b**) Neusilin®US2; (**c**) MCM-41; (**d**) SBA-15; and (**e**) SBA-15_LP.

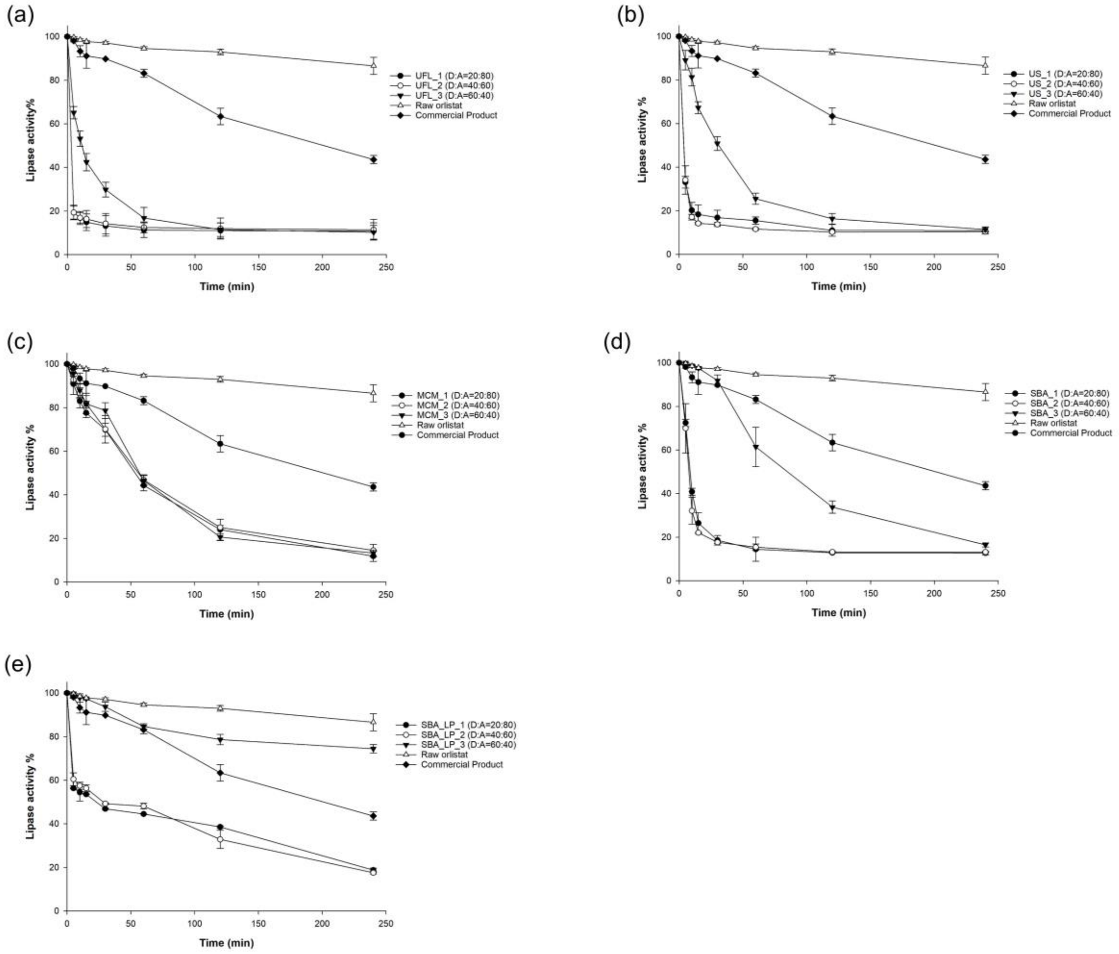

Figure S2. Inhibition of lipase by orlistat loaded onto mesoporous silica at three different drug loading ratio prepared using SCMA process: (**a**) Neusilin®UFL2; (**b**) Neusilin®US2; (**c**) MCM-41; (**d**) SBA-15; and (**e**) SBA-15_LP.
